# Adipose-derived mesenchymal stem cells formed acinar-like structure when stimulated with breast epithelial cells in three-dimensional culture

**DOI:** 10.1371/journal.pone.0204077

**Published:** 2018-10-18

**Authors:** Jing Tong, Shan Mou, Lingyun Xiong, Zhenxing Wang, Rongrong Wang, Annika Weigand, Quan Yuan, Raymund E. Horch, Jiaming Sun, Jie Yang

**Affiliations:** 1 Department of Plastic Surgery, Union Hospital, Tongji Medical College, Huazhong University of Science and Technology, Wuhan, China; 2 Department of Plastic and Hand Surgery and Laboratory for Tissue Engineering and Regenerative Medicine, University Hospital Erlangen, Friedrich Alexander University, Erlangen‐Nuernberg, FAU, Germany; Università degli Studi della Campania, ITALY

## Abstract

Lipotransfer has been applied in breast augmentation surgery for several years and the resident adipose-derived stem cells (ASCs) play an important role in enhancing fat graft survival. However, the interaction between ASCs and mammary epithelium is not fully understood. Many studies have shown that ASCs have a tumor-supportive effect in breast cancer. To the best of our knowledge, this is the first study on the effect of mammary epithelial cells on the human ASCs in 3D culture. ASCs were cultivated on matrigel in the conditioned medium (CM) prepared from a human breast epithelial cell line (HBL-100). The ASCs formed *KRT18*-positive acini-like structures after stimulation with breast epithelial cells. The expression of epithelial genes (*CDH1* and *KRT18*) was up-regulated while the expression of mesenchymal specific genes (*CDH2* and *VIM*) was down-regulated as determined by qRT-PCR. The stemness marker (CD29) and angiogenic factors (CD31 and VEGF) were also down-regulated as examined by immunofluorescence. In addition, the CM obtained from HBL-100 enhanced the migration and inhibited the adipogenic differentiation of ASCs. These results demonstrate that ASCs have the ability to transform into epithelial-like cells when cultured with mammary epithelial cells. Given these observations, we infer that ASCs have a positive effect on lipotransfer, not only due to their ability to secrete growth factors, but also due to their direct participation in the formation of new breast tissue.

## Introduction

Breast augmentation with lipotransfer has recently become one of the most frequently demanded operations in aesthetic surgery. However, characteristics of the recipient area, the processing of the lipotransplant tissue conditions in the breast, and local angiogenesis have been found to affect the outcome of lipotransfer. In addition, from a technical point of view fat transfer is limited by the unpredictable and variable post operational absorption rate [1].

Cell-assisted lipotransfer (CAL) is a new and widely used technology that improves the long-time stability of free fat transfer by increasing the number of adipose-derived stem cells (ASCs) which are key determinants of the outcome of lipotransfer [2]. ASCs are heterogeneous mesenchymal stem cells characterized by multiple markers and have the ability to differentiate into cells of all germ layers [3, 4]. ASCs can be harvested from lipoaspirates with a higher yield and a lower local morbidity compared to mesenchymal stromal cells from bone marrow [5]. Injury, inflammation, and even cancer microenvironment can increase the secretion of ASCs [6]. After lipofilling, only those cells on the outer layer of the transplant with adequate vascularization can survive. Nearly all other injected cells undergo apoptosis and death within weeks. They are eventually replaced by the differentiating ASCs or through the recruitment of local autologous cells [7, 8]. Koellensperger et al. found that the injected ASCs can be redetected throughout the follow-up period of one year at the injection site within the dermal and subcutaneous layers [9]. Yet, how long ASCs from lipotransfer can survive in a normal breast tissue is still unknown. Breast tissue is unique from other gland tissue is that the breast epithelium continues to develop after birth and undergoes extended remodeling with cycles of branching, acini formation, and dissolution during puberty, pregnancy, lactation and menstrual cycles. Little is known about the long-term outcomes of the interaction between ASCs and mammary epithelium [10, 11].

In this study, ASCs were isolated from abdominal fat tissue. The influence of the nonmalignant breast epithelial cells HBL-100 on ASC was studied in a 3D environment prepared with matrigel. As a well-known commercial product made from Engelbreth-Holm-Swarm (EHS) extracts, matrigel is a common matrix for the 3D culture models of normal and malignant breast epithelial cells [12, 13]. The aim of the present study was to investigate cell-cell interactions between ASC and mammary epithelial cells to gain deeper insights into the supportive role of ASC in lipotransfer.

## Materials and methods

This study was reviewed and approved by the Research Ethics Committee of Huazhong University of Science and Technology. Our Institutional Review Board waived the need for informed consent. The study protocol conducted in accordance with the Declaration of Helsinki. HBL-100 cell line (CXO129HBL-100480) was purchased from Boster Biotech (Wuhan, China). MCF-7 was purchased from the Shanghai Cell Bank of the Chinese Academy of Science.

### HBL-100 and media conditioning

The HBL-100 cell line was cultured in DMEM/F12 supplemented with 10% FBS. Conditioned medium (CM) was collected when the cultured HBL-100 cells reached 70% confluence HBL-100 after 24 hours. After centrifugation and filtration through a 0.22 μm filter to remove the cells, the medium was stored at -80°C. The CM was thawed at 4°C overnight and was supplemented with 2% matrigel (Growth Factor Reduced; BD Biosciences, CA, USA) before used. DMEM/F12 containing 10% FBS was used as a control.

### Isolation and identification of ASCs

The patients (n = 5) were enrolled using the following criteria: no primary disease, age between 20 and 30 years, and no liposuction history. ASCs were harvested from the adipose tissue of five patients undergoing abdomen liposuction between August 2014 and January 2015. The isolation procedure was performed according to our previous studies [14, 15]. Briefly, the aspiration was washed with PBS and digested at 37°C with 0.1% collagenase I (Sigma-Aldrich, St. Louis, USA) for 30 minutes. The stromal-vascular fraction was separated by centrifugation at 1,200 x g for 10 minutes. The cell pellet was resuspended in DMEM/F12 (Invitrogen, Carlsbad, CA, USA) supplemented with 10% FBS, 100 units/mL penicillin and 0.1 mg/ml streptomycin (Sigma-Aldrich, St. Louis, MO, USA). The medium was replaced each day. The third-passage ASCs were used. Multipotency of ASCs was assessed by adipogenic, osteogenic and chondrogenic differentiation. Flow cytometry was used to characterize the biomarkers of the isolated ASCs.

Adipogenic differentiation was induced by culturing ASCs for 3 weeks in the adipogenic medium (HUXMD-90031; Cyagen Biosciences, Guangzhou, China) which contained DMEM supplemented with 10% FBS, 1 mM dexamethasone, 1 μg/mL insulin, 200 μM indomethacin, 0.5 mM isobutylmethylxanthine, 100 U/mL penicillin, and 100 mg/mL streptomycin). The differentiated cells were stained with Oil Red O (Sigma-Aldrich, St. Louis, USA). Osteogenic differentiation was induced by culturing ASCs for 3 weeks in a osteogenic medium (HUXMA-90021; Cyagen Biosciences; Guangzhou, China) which contained DMEM supplemented with 10% FBS, 10 nM dexamethasone, 10 mM β-glycerophosphate, 10 nM 1,25-(OH) 2 vitamin D3, 0.15 mM ascorbate-2-phosphate,100 U/mL penicillin, and 100 mg/mL streptomycin. The differentiated cells were stained with Alizarin Red (Sigma-Aldrich, St. Louis, USA). Chondrogenic differentiation was induced by culturing ASCs for 3 weeks in Chondrogenic medium (HUXMD-9004; Cyagen Biosciences, Guangzhou, China) which contained DMEM supplemented with 1% FBS, 6.25 μg/mL insulin, 10 ng/mL TGF-beta1, and 50 nM L-ascorbic-acid-2-phosphate. The differentiated cells were stained with Toluidine Blue O (Sigma-Aldrich, St. Louis, USA).

Cells for flow cytometry analysis were harvested by trypsinization in PBS and incubated with antibodies CD90-FITC, CD73-APC, CD105-PE-Cy7, CD34-PE, CD45-PorCP-Cyanino and CD14-APC-eFluor (eBioscience, CA, USA) separately at 4°C for 40 minutes. Cells incubated without antibodies were used as controls. Thereafter the mixture was centrifuged, re-suspended in PBS and analyzed using a flow cytometer (BD Biosciences, Franklin Lakes, NJ). Data acquisition and analyses were performed by FACSDiva software (BD Biosciences, Franklin Lakes, USA).

### Cell proliferation assay

The wells were coated with matrigel at 37°C for 30 minutes and was used for 3D culture. The wells without matrigel were used for 2D culture. ASCs were harvested and divided into four groups: which were ASC-3D, HASC-3D, ASC-2D and HASC-2D. HASC are ASCs that were cultured in a conditional medium obtain from HBL-100. ASCs for 3D culture were resuspended in DMEM/F12 supplemented with 10% FBS and 2% matrigel. ASCs for 2D culture were resuspended in DMEM/F12 supplemented with 10% FBS. The cells were seeded in the wells (2 × 10^4^ /cm^2^) and cultured for 12 hours before changing the medium to CM or control medium. On day 1, 3, 5 and 7, the cck-8 assay was performed according to the manufacturer’s instruction (CCK-8, Beyotime Biotechnology, China). Cells were incubated with CCK-8 for 3 hours and the absorbance was measured at 450nm. Each measurement was performed in triplicate and the experiments were repeated three times. ASCs cultured in the conditioned medium of HBL-100 were referred to HASCs.

### Cell migration assays

HBL-100 cells (2.5 × 10^5^ /well) suspended in DMEM/F12 containing 10% FBS were seeded on a 24-well plate. MCF-7 cells were used as positive control and DMEM/F12 containing 10% FBS without any cells was used as the negative control. After 12 hours, ASCs (1.5 × 10^5^/well) suspended in DMEM/F12 were seeded on the upper chamber (transwell 3422; Corning, America) and the media was replaced with DMEM/F12 without FBS. After incubation at 37°C for 24 hours, the cells on the upper chamber were removed with cotton swabs. The cells that migrate through the membrane were fixed and stained with 0.1% crystal violet solution. The cell numbers were counted in six random fields for each chamber under a microscope. The experiments were repeated three times.

### Adipogenic differentiation

After 14-days of CM treatment, the adipogenic differentiation of ASCs was re-assessed. ASCs cultured without CM for 14-days were used as a control. Cells were stained with Oil Red O. The lipid droplets were photographed using a microscope. Stained lipid droplets were extracted with isopropanol and absorbance was measured at 540 nm for quantification.

### Three-dimensional culture system and light microscopic analysis

Each well of an 8-well slide was coated with matrigel, and incubated at 37°C. ASCs (10^4^ cells /mL) were resuspended in DMEM/F12 supplemented with 10% FBS and 2% matrigel. The cell suspension was added to each well (400 μL/well) and cultured for three days. Then, the cells were subjected to serum starvation in DMEM/F12 without FBS for 12 hours. The media was changed to corresponding CM or control media and cells were incubated for 12 days. ASCs were observed on a light microscope at the time-interval of 3 hours on the first three days and once a day on the following 12 days after induction by CM.

### Real time-PCR

Total RNA was isolated from ASCs with Trizol Reagent on day12 of indirect coculture with HBL-100 cells. Reverse transcription was performed according to the manufacturer’s protocol using First Strand cDNA Synthesis Kit (TOYOBO, Osaka, Japan). Quantitative RT-PCR was carried out by Step One™ Real-Time PCR System and THUNDERBIRD SYBR qPCR Mix (TOYOBO, Osaka, Japan). The 2^-ΔΔCt^ formula was used to calculate the relative gene expression between samples. Primers used are listed in Table 1. Each measurement was performed in triplicate and the experiment was repeated three times using ASCs from three patients. *ACTB* was used as the housekeeping gene.

**Table 1:** 

Target gene	Primer type	Sequence
***VIM***	Forward Reverse	5’-TCAGAATATGAAGGAGGAAATAAC-3’ 5’-GAGTGGGTATCAACCAGAGGGAGT-3’;
***CDH2***	Forward Reverse	5’- CCCCTCAAGTGTTACCTCA-3’, 5’-AAATCACCATTAAGCCGAGT-3’
***KRT18***	Forward Reverse	5’-TTGACAATGCCCGTCTTGCT-3’ 5’- GCCTTTTACTTCCTCTTCGTGGT-3’
***CDH1***	Forward Reverse	5’-CTTTGACGCCGAGAGCTAC -3’ 5’-TTTGAATCGGGTGTCGAGGG -3’
***ACTB***	Forward Reverse	5’- GTCCACCGCAAATGCTTCTA-3’ 5’- TGCTGTCACCTTCACCGTTC-3’

### Immunofluorescence

The whole 3D culture systems were fixed with 2% paraformaldehyde permeabilized with 0.5% Triton x-100 and blocked with 5% BSA. Samples were incubated over night at 4°C with a mouse anti-human antibody against human cytokeratin 18 (1:200; GENE Tex, CA, USA), cytokeratin 8 (1:100; Abcam, Cambridge, UK) CD29 (1:100; Abcam, Cambridge, UK), CD90 (1:100; Abcam, Cambridge, UK), CD31 (1:200; Abcam, Cambridge, UK), VEGF (1:200; Abcam, Cambridge, UK) and DAPI (Invitrogen, CA, USA). Breast tissue harvested from a healthy woman undergoing reduction mammoplasty was used as a positive control. The images were acquired with a fluorescence microscope (Olympus, Tokyo, Japan).

## Results

### Isolation and characterization of ASCs

**Fig 1:**
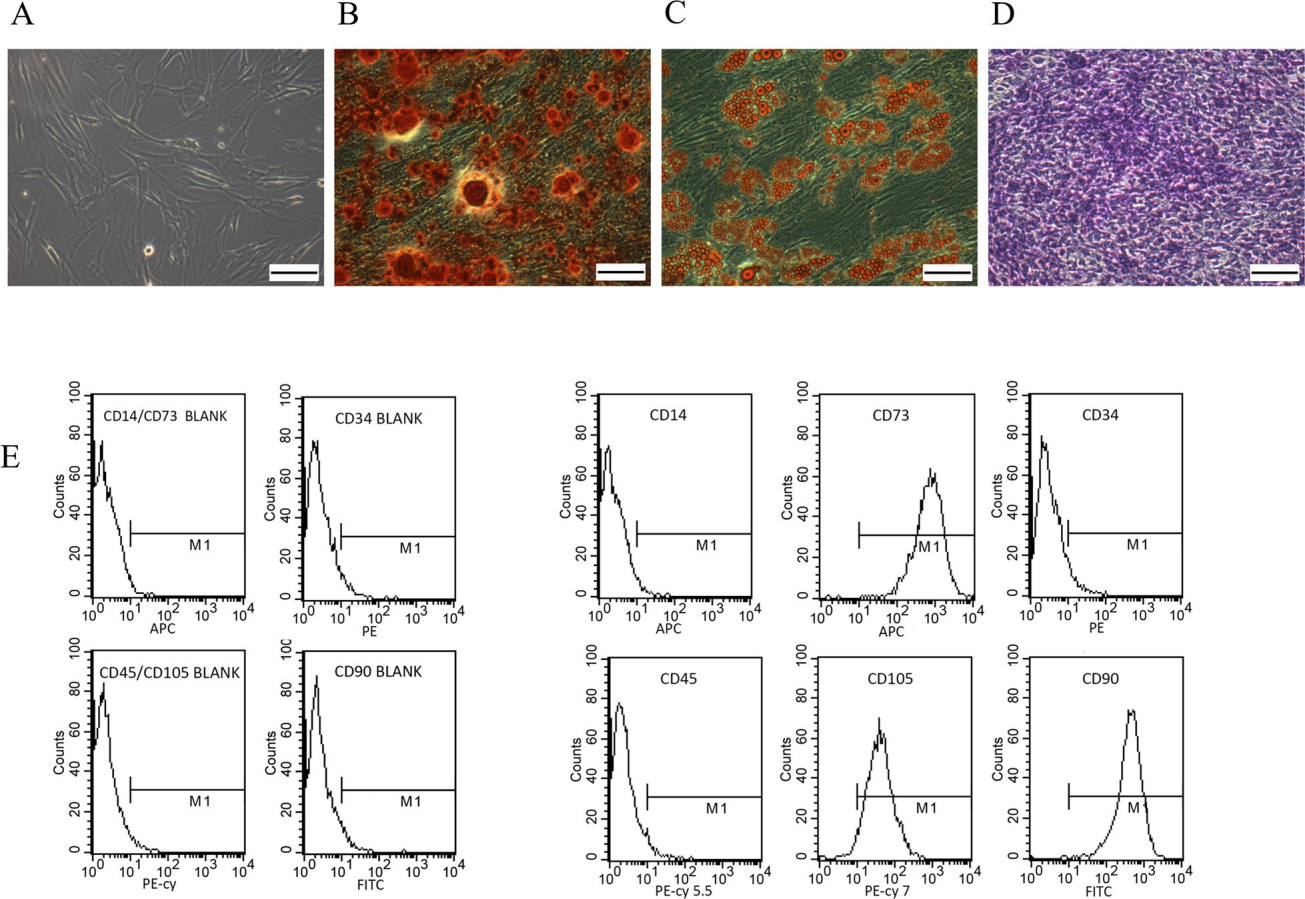
**Multi-differentiation (adipogenic differentiation, osteogenic differentiation and chondrogenic differentiation) of human adipose derived stem cells (ASCs) and surface markers of ASCs assessed by flow cytometry A:** Morphological observation of ASCs under an inverted light microscope. **B:** Alizarin Red staining of osteogenic differentiation on 21st day. **C:** Oil Red O staining of adipogenic differentiation on 21st day. **D:** Toliudine Blue staining of chondrogenic differentiation. **E:** Positive and negative surface markers of ASCs determined by flow cytometry: ASCs expressed CD105, CD73 and CD90, and lack expression of CD45, CD34, and CD14. Scale bar = 100μm (A-D).

ASCs were identified by analysis of adipogenic differentiation with Oil Red O staining, osteogenic differentiation with alizarin red staining, chondrogenic differentiation with Toluidine Blue staining and analysis of distinct surface markers using flow cytometry (Fig 1).

Compared to undifferentiated ASCs group (Fig 1A), extracellular calcium was revealed by alizarin staining in the osteogenically differentiated ASCs (Fig 1B). There were lipid drops inside the cytoplasm of the adipogenically differentiated ASCs as demonstrated by Oil Red O staining (Fig 1C). The chondrogenic differentiation of ASCs was confirmed by Toluidine Blue staining (Fig 1D). Flow cytometry analysis showed the expression of surface markers such as CD105, CD73, and CD90 confirming the stemness and mesenchymal features of the cells. In contrast, there were few CD34, CD14 and CD45-positive cells suggesting that there were few endothelial progenitors and hematopoietic cells (Fig 1E). These findings showed that the cells isolated met the minimal criteria to be defined as ASCs [14].

### Changes in proliferation, migration ability, cell morphology and adipogenic differentiation

**Fig 2:**
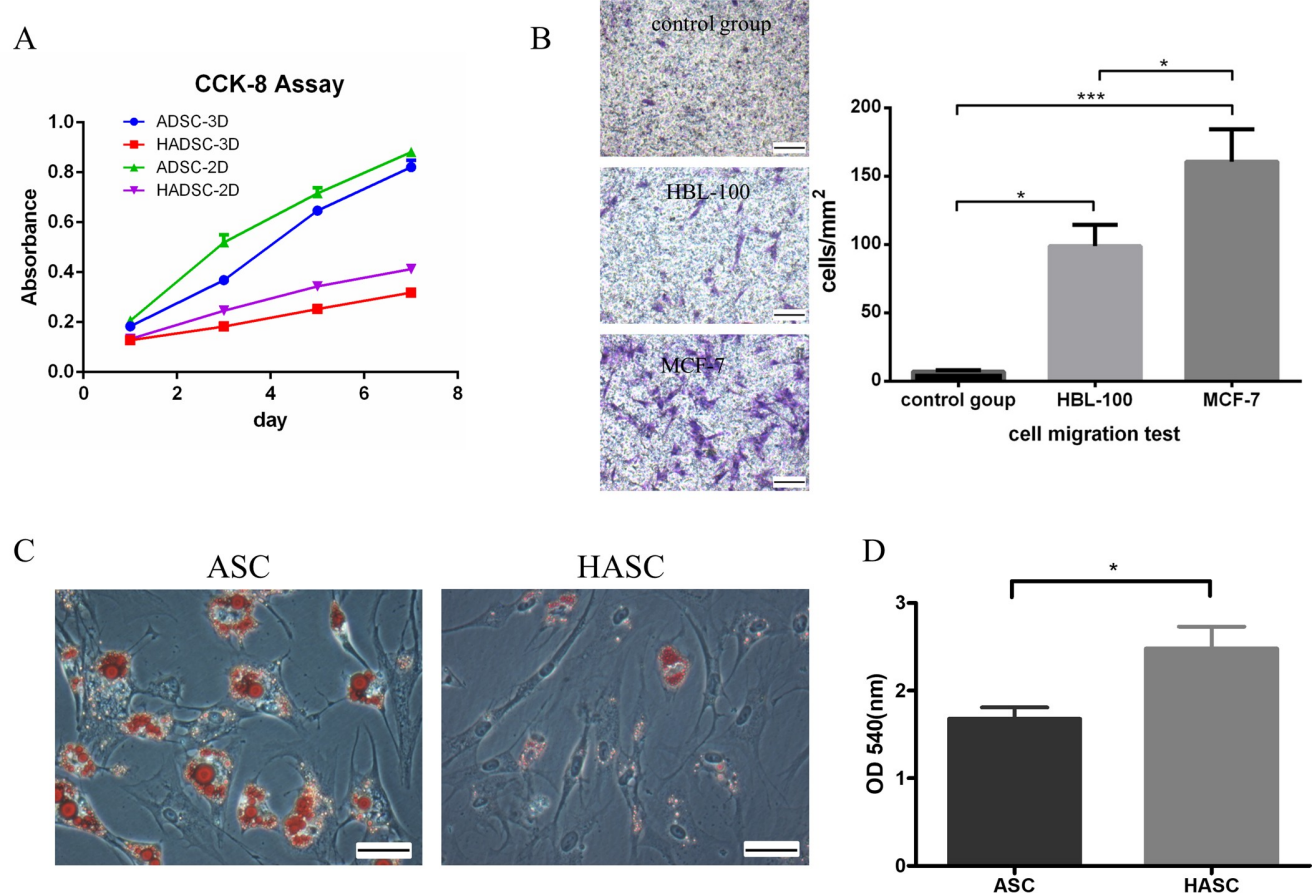
Changes of ASCs in 3D culture. **A:** CCK8 assay. The y-axis shows the amount of absorbance, the y-axis the measuring time points. **B:** The Transwell migration assay results. The y-axis shows the cells number per mm2 and the x-axis shows the different groups. * P<0.05, ***P<0.001, n = 3. Scale bar = 40μm. **C:** ASCs and HASCs were underwent 21 days of adipogenic differentiation. The cells were fixed and stained with Oil-Red-O. The images were took by a microscope. Scale bar = 50μm. **D:** Quantitative analysis of Oil Red O staining. Dye stained cells was extracted with isopropanol and the absorbance at 540 nm was measured. * P<0.05, ***P<0.001, n = 3.

CCK-8 assays showed that both three-dimensional culture and conditioned medium reduced the proliferation of ASCs (Fig 2A). According to the results of the cell migration assay, HBL-100 significantly promoted the migration ability of ASCs compared to negative control (98.94 ± 4.50 vs. 6.91±1.02 cells/mm^2^; P<0.05; Fig 2B). The effect of HBL-100 was significantly weaker compared to that of MCF-7 (98.94 ± 4.50 vs. 160.70 ±6.87 cells/mm^2^; P<0.05; Fig 2B).

Adipogenic differentiation of both ASCs and HASCs was confirmed by positive Oil-Red-O staining after 14 days of culture in the differentiation media. CM from HBL-100 was found to inhibit the formation of lipid droplets compared with ASCs (Fig 2C and 2D; P<0.05).

**Fig 3:**
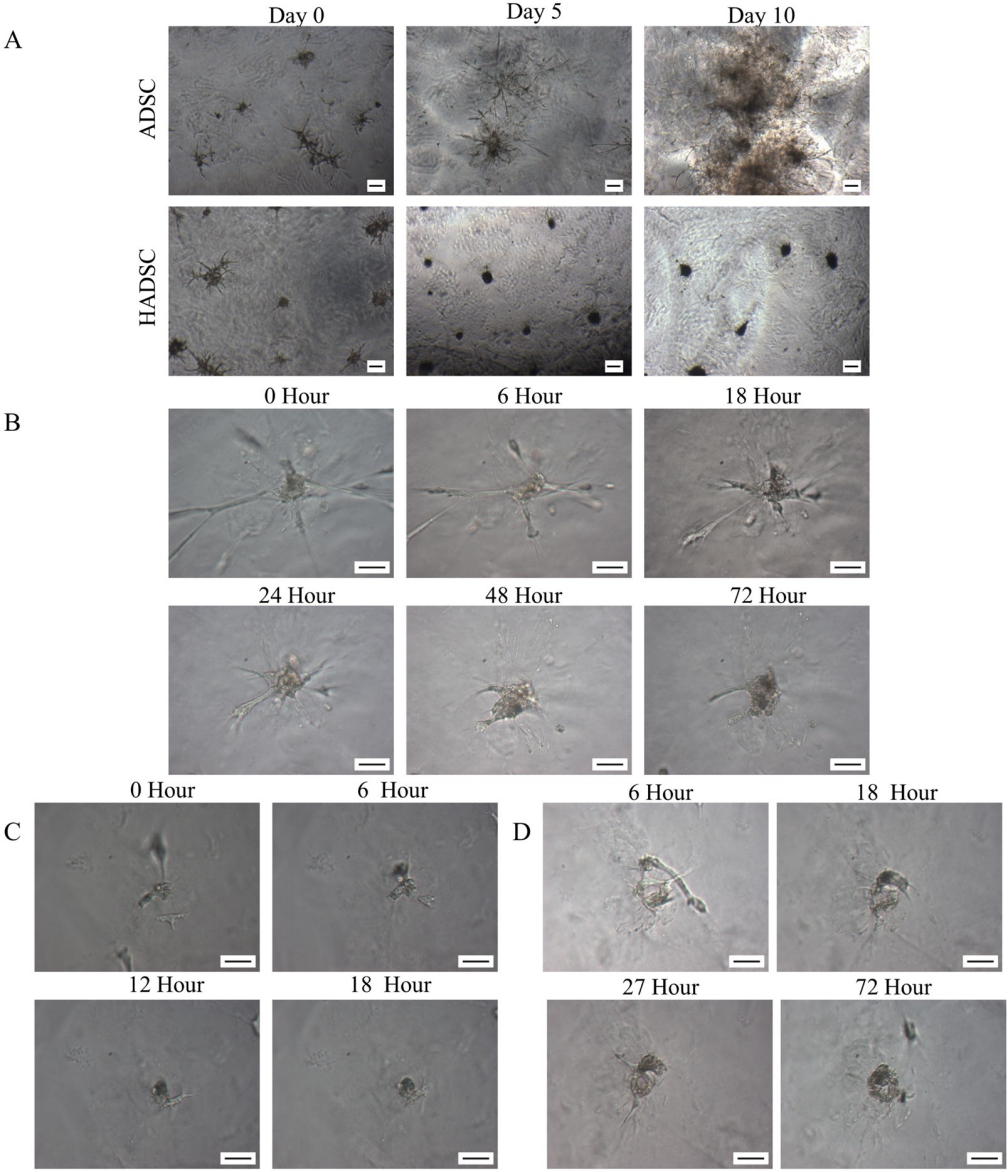
Different behaviors of ASCs induced by HBL-100 conditioned medium in 3D culture. **A:** The Day 0 referred to the day in which ASCs had undergone serum starvation. In the control group (ASC), the branching structure formed by ASCs continued growing on day 5 and formed a network-like structure on day 10. In the conditional medium group (HASCs), the branching structure formed an acinar-like structure on day 5 which failed to grow larger on day 10. **B:** Cell contraction. **C:** Cell assembly. **D:** Spinal Cell Contraction. Scale bar = 40μm (A-C).

When cultured on the growth factor-reduced matrigel, ASCs assembled into sphere-like structures at 24 hours and then formed branched structures as early as 2 days in a culture which finally became a branch-like sphere network (Fig 3A). The network structures formed by ASCs grew larger and spread over the gel. When treated with breast epithelial cell conditioned medium, the stretched ASCs tended to contract (Fig 3B) and the nearby cells tended to form colonies (Fig 3C). The contraction and colony-formation continued up to 72 hours. Interestingly, the spiral multi-cell structure formed a ring-like structure after HBL-100 induction (Fig 3D). The spherical structure failed to grow larger and many of them had thick finger like protrusions. The shape and size of ASCs cell on the matrigel before induction affected the shape and size of HASCs cell groups. However, in the corner of the same chamber HASCs which directly grew on the surface of the chamber slide showed a significantly different stretched morphology and continue to grow.

### Quantitative real-time PCR analysis of the three-dimensional cultures

**Fig 4:**
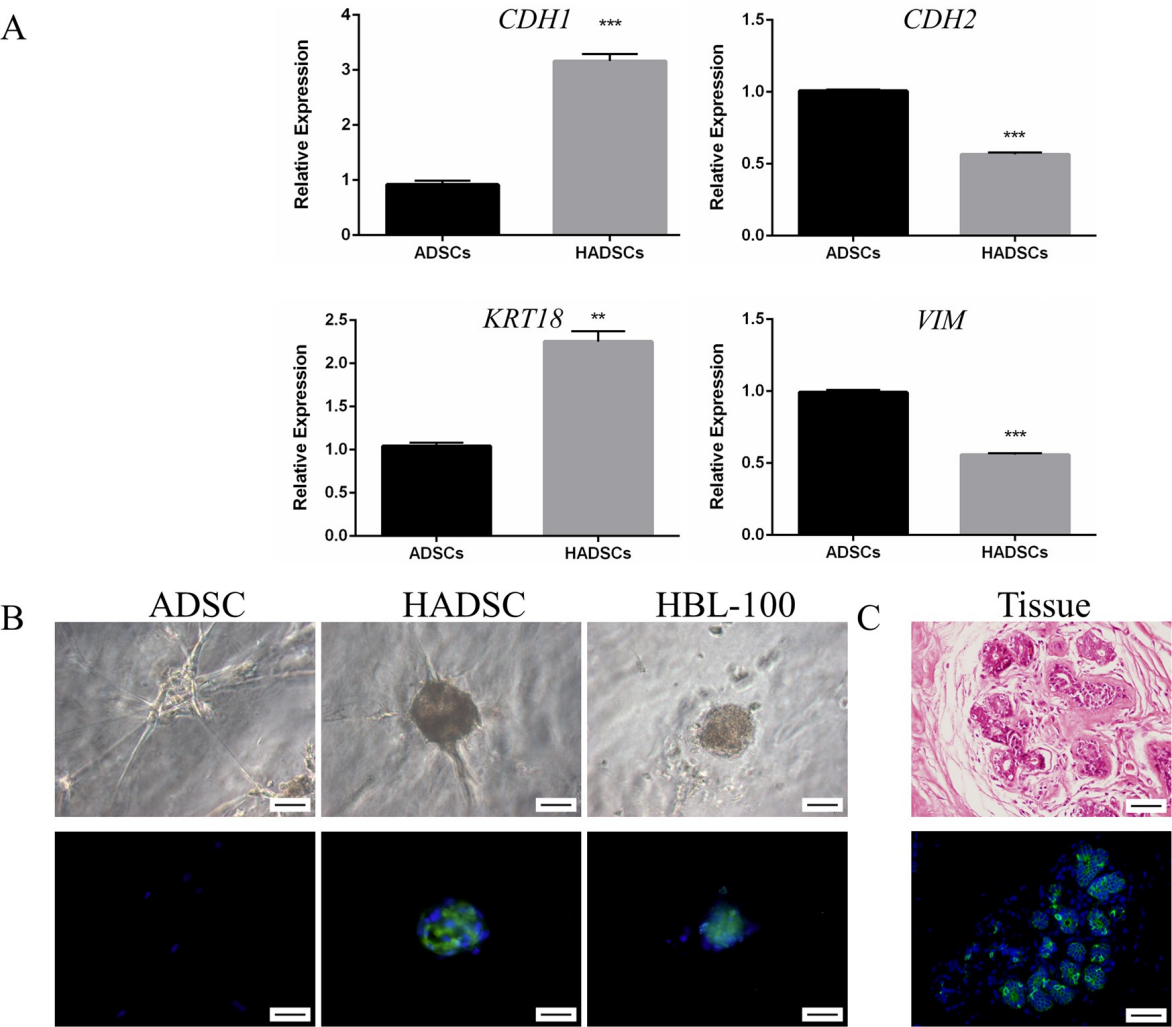
KRT18 expression and mRNA expression of ASCs induced by HBL-100 conditioned medium in 3D culture. **A:** The relative expression of epithelial specific genes *CDH1*, *KRT18* was significantly higher in HASCs compared with ASCs while the relative expression of mesenchymal specific genes *CDH2*, *VIM* was significantly lower. MRNA expression was measured by quantitative RT-PCR. 2^-△△^ CT method was used to calculate relative expression of genes. * * P<0.01, *** P<0.001, n = 3. **B:** *KRT18* (green) expression in 3D culture. Nuclei were stained with DAPI (blue). **C:** Human mammary tissue was observed with HE staining. *KRT18*-specific antibody was used to mark human mammary epithelial cells or epithelial-like cells. Scale bar = 40μm (B and C).

Total RNA was isolated from the ASCs of different groups. Relative expression of epithelial and mesenchymal-specific genes was assessed using RT-qPCR analysis with specific primers (Table 1). The relative expression of *CDH1* was significantly higher in HASCs compared with ASCs (3.16 ± 0.07 vs. 0.92 ± 0.04; P<0.01; Fig 4A). The relative expression of *KRT18* was significantly higher in HASCs compared with ASCs (2.25 ± 0.12 vs. 1.04 ± 0.04; P<0.01; Fig 4A). The relative expression of *CDH2* was significantly lower in HASCs compared with ASCs (0.57 ± 0.01 vs. 1.01 ± 0.01; P<0.01; Fig 4A). The relative expression of *VIM* was significantly lower in HASCs compared with ASCs (0.56±0.01 vs. 1.00±0.02; P<0.01; Fig 4A).

### Immunofluorescence

**Fig 5:**
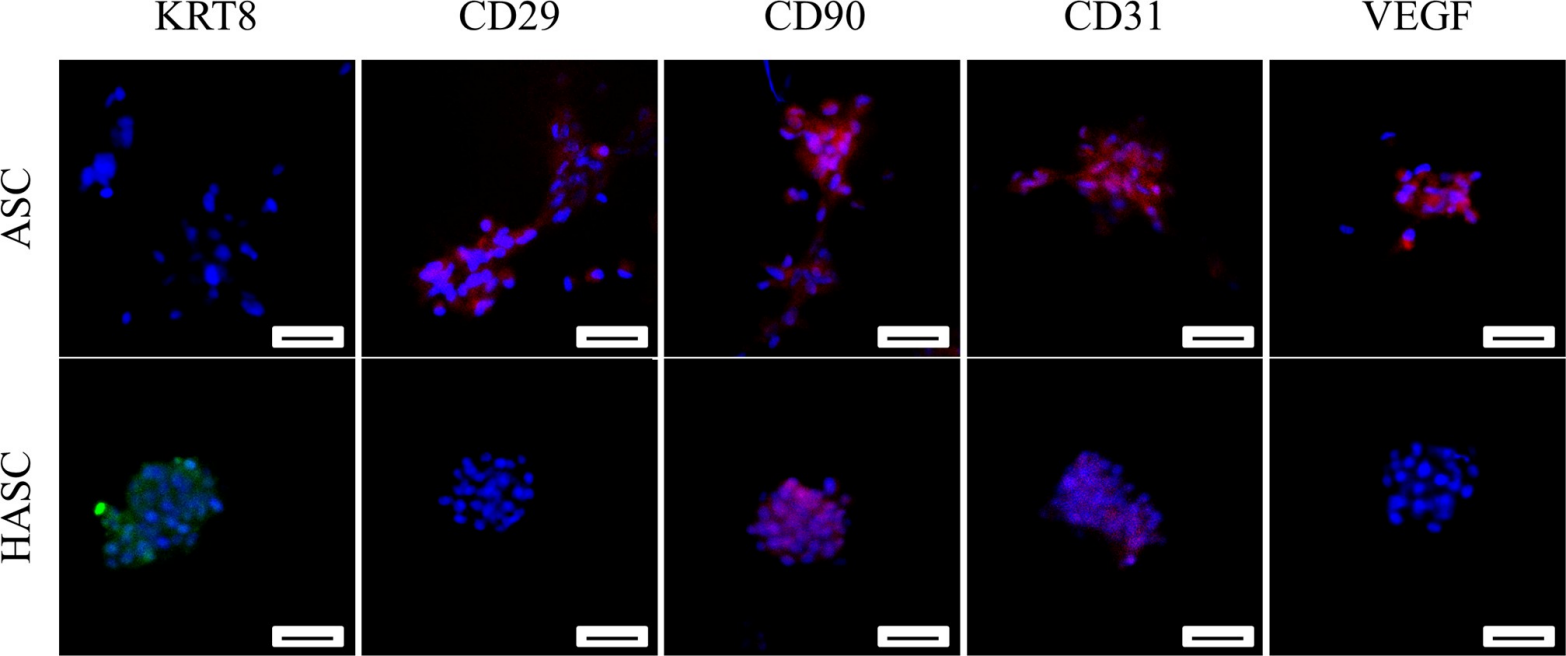
Analyses of stem cell markers and angiogenic factors expression by immunofluorescence showing CM downregulated the stemness and angiogenic differentiation of ASCs in 3D. Representative fluorescence images of ASCs and HASCs stained for KRT8, stem cell markers (CD29, CD90) and angiogenic factors (CD31, VEGF). Nuclei were stained with DAPI (blue). Scale bar = 20μm.

In the control group, *KRT18* expression was not detected in ASCs and showed branched growth. In the conditioned medium group, ASCs formed acinar-like structures with high expression of *KRT18* (Fig 4B). These structures resembled acinar structures formed by HBL-100 in 3D (Fig 4B). The human breast tissue that was used as a positive control had alveolar structures with high expression of *KRT18* (Fig 4C). HASCs also expressed another epithelial marker *KRT8*. The Stemness marker (CD29) and angiogenic factors (CD31, VEGF) were also found down-regulated by immunofluorescence (Fig 5). No change was detectable for another stemness marker (CD90).

## Discussion

Patients undergoing lipotransfer frequently require repeated injection in the breast to achieve satisfying appearance. To address this problem, clinicians attempt to increase the amount of ASCs in lipografts using filtration, centrifugation or rough purification [15]. In addition, ex-vivo expanded ASCs have been used to increase graft viability [16]. However, the interaction between ASCs and mammary epithelium has not been sufficiently clarified. In fact, there is an ongoing debate on the potential side effects of a direct interaction between ASC and breast glandular cells [17, 18].

When lipotransfer was first introduced in breast surgery, interactions between ASCs and mammary epithelial cells especially malignant epithelial cells became an important issue. Clinical studies on CAL for aesthetic breast augmentation focused mainly on efficiency, complications and risk of microcalcifications. A match cohort study evaluating the oncological safety of fat grafting was performed at the European Institute of Oncology [19]. A higher risk of cancer recurrence was reported after lipotransfer in patients who had been diagnosed with intraepithelial malignancies. The evidence from most studies on the oncological safety of fat grafting for breast reconstruction is questionable because of the lack of matched control groups, small population sizes and short follow-ups [11, 20, 21]. Currently, there is limited knowledge on the long-time safety of injected exogenous ASCs into the breast tissue. ASCs are thought to exert tumor -supportive effects, but there is no evidence that they are directly tumorigenic [6]. A crosstalk mediated by HGF/c-MET between ASCs and breast cancer cells increased the invasion ability of cancer cells and their growth rate [22]. Cytokines and chemokines secreted by ASCs support the formation of an microenvironment favorable for neoangiogenesis and inflammation which further recruits more ASCs. ASCs can also induce epithelial-to-mesenchymal transition (EMT), increase chemo sensitivity and angiogenesis of human breast cancer cells [22–26]. Moreover, ASCs favor tumor angiogenesis and increase tumor aggressiveness. On the other hand, cancer cells including those of epithelial origin promote the proliferation of ASCs and maintenance of stemness and stability of ASCs which in turn create a stem-rich microenvironment that favors the survival and growth of tumor[27]. In patients affected with a pre-existing cancer, lipotransfer especially CAL can be a risky approach.

It has been recognized that the tissue microenvironment influences the stem cells behavior [28–30]. Adult mouse spermatogenic cells, neural stem cells and human embryonic carcinoma cells were found to differentiate into mammary epithelial cells dependent on mouse breast microenvironment [28–30]. Grafts of adipose tissue from different sites of the body of adult mice gave rise to milk-secreting epithelial cells when exposed to the mammary gland microenvironment in pregnant and lactating females [31]. ASCs can undergo epithelial differentiation when co-cultured with mature epithelial cells like hepatic cells [32]. In our previous study, we demonstrated that ASCs express the epithelial cell marker *KRT18* when stimulated by the mammary epithelial cell in 2D [33]. However, according to Chai et al., a range of epithelial and mesenchymal markers are expressed both in epithelial and mesenchymal cells. Some epithelial markers, such as *CLDN5*, *OCLN*, *DSG1* and *TJP1* were highly expressed in fibroblasts than in epithelial cells [29].

In tissue microenvironment, the extracellular matrix (ECM) is an important factor for cell differentiation. Desiderio at al. found that NG2(+) ASCs on cross-linked hyaluronic acids can produce a human skeletal muscle tissue in vivo without the need for a pre-differentiation step in vitro[34]. In another study, Ferraro et al. found that ASCs loaded on a collagen scaffold can differentiate in vivo into both adipose and loose connective tissues[35].

Extracellular matrix in the mammary gland is important for gland development and remodeling, and influences the fate of epithelial cells. In the breast, cell-cell and cell-extracellular matrix interactions provide a complex biochemical signaling network which is essential for the development and proliferation of the mammary epithelium [36, 37]. The base membrane (BM) of the mammary gland is a specialized type of ECM which connects epithelial and connective tissues. It is composed of laminins and collagen IV. It also provides the microenvironment of the mammary epithelium with soluble factors. When cells are cultivated as a monolayer in 2D, signals originating from the BM cannot be mimicked in vitro. A 3D culture made of laminins-rich BM can retain these important signals and support physiologically relevant functions in vitro. Matrigel contains laminins and collagen IV and is often used in 3D mammary epithelium studies. In the present study, HBL-100 cells were harvested from the early milk of a healthy woman [38]. This cell line is an appropriate model of the human normal breast as it is a non-tumorigenic cell line at low passages and easy to culture [39].

This study investigated how ASCs are affected by nonmalignant breast cells HBL-100 on a matrigel matrix. ASCs formed an acinar-like structure with high expression of early epithelial cell marker *KRT18* induced by HBL-100 in 3D culture. Interestingly, the expression of epithelial markers (*KRT18* and *CDH1*) was up-regulated while the expression of mesenchymal markers (*CDH2* and *VIM*) was down-regulated.

*VIM* and keratins are found in intermediate filaments the major mechanical stress protectors in the cellular skeleton. They play major roles in cell growth, differentiation and migration. Intermediate filaments exhibit tissue specificity. *VIM* is predominantly expressed in cells of mesenchymal tissue and mesodermal origin. It is also found in migrating epithelium cells. Keratins are generally expressed in the epithelial tissue. There are more than 20 different keratins which can be used to identify the type of epithelium [40]. *KRT18* is one of the keratins commonly expressed in ectodermic epithelium including luminal epithelium [41]. Here, we found that *VIM* mRNA expression was significantly lower and *KRT18* mRNA expression was significantly higher in ASCs after stimulation with CM prepared from HBL-100. Furthermore, KRT18 protein was expressed in the acinar-like structures formed by ASCs.

*CDH1* is considered as a hallmark of epithelium and the major classic cadherin in luminal epithelial cells which is required for lactation, branching morphogenesis, and homeostasis. *CDH2* is expressed in neural tissue and mesothelium [42]. The switch from *CDH2* to *CDH1* expression represents MET which are regulated by transcription factors, such as SNAIL, TWIST and SLUG. TWIST affects cell movement and participate in development whereas SLUG represses epithelial proteins such as cadherins and active cytoskeletal markers such as vimentin. The down-regulation of these factors triggers the MET process[43].

The switched expression from Vimentin and CDH2 to CK18 and CDH1 are considered as hall markers of MET [32]. Our results suggest the change of H-ADSCs was the MET in our study. Mesenchymal-epithelial transition (MET) and epithelial-mesenchymal transition (EMT) are important processes of cell shape and genotype changes during development, organogenesis, tissue repair and disease progression. EMT is the reversal and complementary process of MET. During MET process, mesenchymal cells lose mesenchymal properties and acquire epithelial properties which include acquiring apical-basal polarity, epithelial gene expression formation of adherents or tight junctions. [44].

The expression of stemness marker (CD29) and angiogenic factors (CD31, VEGF) was lower in HASCs compared with ASCs. Moreover, HASCs showed little ability to undergo adipogenic differentiation. Interestingly, according to Paino et al., the breast cancer cell line MCF-7 increased proliferation of ASCs. When co-cultured with MCF-7, the expression of stemness and mesenchymal markers (CD90, CD29, CD324 and vimentin) or the expression of EMT markers in human ASCs were not altered. There is a decrease in angiogenic factors, including CD31, and VEGF, and an increase in stemness gene (Sox2, Nanog and OCT3/4). ASCs retained stem cell properties and can differentated in both adipocytes and endothelial cells when co-cultured with MCF-7. In our study, ASCs showed different behavior from nonmalignant breast cell HBL-100. CM from HBL-100 decreased proliferation and inhibited the adipogenic differentiation of ASCs. There is a decrease in the stemness marker CD29. The switched expression from Vimentin and CDH2 to CK18 and CDH1 indicated that HASCs underwent MET.

Although we found that ASCs can form acinar-like structures in a 3D culture, which also showed a genetic change of MET after HBL-100 induction, it is unlikely that the differentiation is complete. 3D cultures resemble native tissue microenvironment compared to 2D cultures. Results of a study on foreign mammary epithelial cell transplantation showed that fibroblasts of breast tissue are important for mammary epithelium survival and mammary development [45, 46]. Furthermore, it should be noted that hormonal signaling is also required for the conversion of non-mammary mouse cells to mammary cell fate in vivo [47].

## Conclusion

This study found that ASCs formed acinar-like structures and showed characteristics of epithelial differentiation when stimulated by the breast epithelial cell line HBL-100 in 3D. In the clinical context, the findings show that these features of ASCs are beneficial for cell-assisted lipotransfer (CAL) for breast reconstruction as they can promote mammary gland growth. However, there are safety concerns regarding the use of CAL for breast reconstruction due to the possible interactions between ASCs and cells in the mammary tissue.

Therefore, in addition to the need to study the effects of the interaction between exogenous ASCs and breast epithelium, the clinical use of ASCs in breast reconstruction especially ASCs that are expanded in vitro, should be given careful attention. It is also recommended that injection of fat graft directly into breast glandular tissue should be avoided until the interaction between ASC and breast glandular cells is fully understood. Until now there is no sufficient data on the safety of CAL to the breast. Hence, we suggest that patients at high-risk for breast cancer should be carefully selected for breast augmentation with lipotransfer. In conclusion, we strongly recommend further clinical investigations on the long-term safety of breast lipotransfer.
